# Multivariable Discriminant Analysis for the Differential Diagnosis of Microcytic Anemia

**DOI:** 10.1155/2013/457834

**Published:** 2013-09-04

**Authors:** Eloísa Urrechaga, Urko Aguirre, Silvia Izquierdo

**Affiliations:** ^1^Laboratorio, Hospital Galdakao-Usansolo, 48960 Galdakao, Vizcaya, Spain; ^2^Unidad de Investigación CIBER Epidemiología y Salud Pública, 48960 Galdakao, Vizcaya, Spain; ^3^Genética Clínica, Servicio de Bioquímica Clínica, Hospital Universitario Miguel Servet, 50009 Zaragoza, Spain

## Abstract

*Introduction*. Iron deficiency anemia and thalassemia are the most common causes of microcytic anemia. Powerful statistical computer programming enables sensitive discriminant analyses to aid in the diagnosis. We aimed at investigating the performance of the multiple discriminant analysis (MDA) to the differential diagnosis of microcytic anemia. *Methods*. The training group was composed of 200 **β**-thalassemia carriers, 65 **α**-thalassemia carriers, 170 iron deficiency anemia (IDA), and 45 mixed cases of thalassemia and acute phase response or iron deficiency. A set of potential predictor parameters that could detect differences among groups were selected: Red Blood Cells (RBC), hemoglobin (Hb), mean cell volume (MCV), mean cell hemoglobin (MCH), and RBC distribution width (RDW). The functions obtained with MDA analysis were applied to a set of 628 consecutive patients with microcytic anemia. *Results*. For classifying patients into two groups (genetic anemia and acquired anemia), only one function was needed; 87.9% **β**-thalassemia carriers, and 83.3% **α**-thalassemia carriers, and 72.1% in the mixed group were correctly classified. *Conclusion*. Linear discriminant functions based on hemogram data can aid in differentiating between IDA and thalassemia, so samples can be efficiently selected for further analysis to confirm the presence of genetic anemia.

## 1. Introduction

Iron deficiency anemia (IDA) and *β*-thalassemia are the most common causes of microcytic anemia.

The differentiation between IDA and microcytosis due to genetic cause has important clinical implications. As all chronic diseases, prevention is important in the overall management of the disease: an appropriate screening, detection of patients, and counsel of couples at risk are the most important procedures for the reduction of morbidity and mortality of the patients [1].

The presumptive identification of hemoglobin disorders must rely on inexpensive methods of detection, to allow an efficient use of the resources: a good method for screening can help, allowing selection of samples for further analysis to confirm the disease.

Definitive methods for diagnosis of thalassemia trait include quantitative analysis of HbA_2_ and DNA studies for specific deletions and mutations. Increased HbA_2_ is considered to be confirmatory for *β*-thalassemia trait. Low or normal values and no evidence of iron deficiency suggest *α*-thalassemia; definitive diagnosis requires molecular methods to detect gene deletions. While being accurate, these tests are too expensive for initial mass screening [2].

The availability of computer, robotic systems, and powerful statistical software has expanded the accessibility of sophisticated statistical analysis. These include analyses employing multiple predictor variables (multivariate analysis) to predict an outcome variable [3]. 

MDA begins with subjects in two or more groups and then uses the discriminant procedure to identify a linear combination of quantitative predictor variables that best characterize the differences among the groups. The discriminant function sums the products of variables multiplied by coefficients. The procedure estimates the coefficients for each variable, and the resulting function can be used to classify new patients.

MDA can be used to develop more sensitive and accurate diagnostic methods for thalassemia detection using the data of the hemogram. We applied stepwise MDA to determinate which red cells derived parameters that are best in differentiating the heritable genetic anemia and the iron deficient state.

The aim of the present study was to investigate the performance of MDA to the differential diagnosis of genetic and acquired microcytic anemia, so samples can be efficiently selected for further analysis to confirm the presumptive diagnosis of thalassemia. 

## 2. Materials and Methods

### 2.1. Criteria for Selecting the Groups of Patients

The study was conducted according to the hospital ethic's guidelines after being approved by the Committee of Ethics and Good Practice of the hospital.

Only adults were included in the present study, and none of them received a transfusion nor had an acute bleeding in the previous month. The samples were obtained in the course of routine analysis, collected in EDTA anticoagulant tubes (Vacutainer Becton-Dickinson, Rutherford, NJ, USA), and run in the analyzers of the LH 1500 Beckman Coulter robotic system (Beckman Coulter Inc., Miami, FL, USA) within 6 hours of collection.

A total of 480 patients were included in the training set, classified into four different disorders: IDA, *α*- and *β*-thalassemia, and a group of thalassemia carriers with other diseases at the moment of the analysis (mixed group).

The IDA group consisted of 170 patients (35.4%), with Hb < 120 g/L, MCV < 80 fL, serum Iron < 7.5 *μ*mol/L, transferrin saturation < 20%, and serum ferritin < 50 *μ*g/L [4]. 

Two hundred *β*-thalassemia carriers (41.6%) and 65 *α*-thalassemia carriers (13.5%), all of them with a previous diagnosis of the disease, were recruited.

A mixed group (9.5%) included 45 thalassemia carriers with acute phase response (APR), iron deficiency, or pregnancy at the moment of the analysis.

Thalassemia screening is routinely performed in our laboratory by means of the measure of their Red Blood Cell parameters. Samples with erythrocytosis (RBC > 5.5·10^12^/L) and microcytosis (MCV < 80 fL) are selected for HbA_2_ quantification (HPLC HA 8160, Menarini Diagnostics, Firenze, Italy). Increased HbA_2_ (>3.5%) is considered to be confirmatory for *β*-thalassemia trait. 

Low HbA_2_ (<2.5%) or a value within the reference range (2.5%–3.5%) is feature of *α*-thalassemia, and these samples are referred for molecular analysis to detect the associated deletions.

Molecular analysis is performed if genetic counsel is required. Molecular characterization of mutations is performed with allele specific oligonucleotide-polymerase chain reaction PCR-ASO techniques [5, 6].

A second group of consecutive patients with microcytic anemia (*n* = 628), extracted from the laboratory workload during the months of January and February 2013, was used as a validation set.

This group consisted of 505 (80.4%) IDA patients, 63 (10.0%) *β*-thalassemia, 16 (2.6%) *α*-thalassemia, and 44 (7.0%) a mixed group of hemoglobinopathies or thalassemia carriers with APR, IDA, or pregnancy at the moment of the analysis: 4 *α*-thalassemia and IDA, 3 *α*-thalassemia and APR; 1 pregnant *α*-thalassemia carrier, 7 *β*-thalassemia and IDA, 5 *β*-thalassemia and APR; 5 pregnant *β*-thalassemia carriers, 10 Hb S, and 4 Hb Lepore; 1 Hb E; 1 Hb C and IDA; 1 Hb S and APR; and 2 pregnant HbS.

### 2.2. Statistical Analysis

A set of potential predictor parameters that could detect differences among the mentioned microcytic anemias were selected: Red Blood Cells (RBC), hemoglobin (Hb), mean cell volume (MCV), mean cell hemoglobin (MCH), and red cells distribution width (RDW).

The outcome of interest was the type of microcytic anemia. It was considered in two different ways: classification type I (IDA, *α*-thalassemia, *β*-thalassemia, and mixed clinical situations) and classification type II (genetic anemia and IDA acquired anemia). 

In the training group, as initial step, an exploratory data analysis of the collected hemogram parameters was performed across the type of disease, using means and standard deviations. To assess mean differences in the mentioned predictor parameters across the different types of disorders according to the classification type I, Kruskall-Wallis nonparametric test for independent samples was used; Wilcoxon nonparametric test was used when classification type II was regarded.

Multivariate discriminant analysis (MDA) was conducted in order to distinguish differences among groups of diseases and to determine how to allocate new observations into the established groups. To this end, the above-mentioned parameters were considered as independent variables whereas the type of disease was the outcome. As the first step, Wilk's Lambda statistic was used to test whether the discriminant model was significant. Moreover, the number of discriminant functions, the corresponding standardized discriminant coefficients, and canonical correlations for each of studied parameters—the ones which maximized the distance between the groups—were also obtained. Correlations higher than 0.40 were considered significant [3].

Thereafter, a classification functional equation was constructed. A case was predicted as being member of the group in which the value of its classification function was the largest. The predicted diagnoses were then compared with the actual diagnoses in each of the original patients. Correct classification was defined as the division between concordant cases (when predicted and actual diagnoses were the same) and the entire sample size. This was performed by means of cross-validation. A scatterplot of the discriminant functions in the training set was depicted. 

The validation set of 628 subjects was employed to evaluate the performance of the classification determined by the established functions in the training group. 

All these statistical analyses where performed for the two mentioned classification types I and II, using the *R* statistical software 2.14 release. A *P* value <0.05 was deemed to be statistically significant. 

## 3. Results

There were significant differences for the disease group distribution in both data sets (*P* < 0.001). 

Table 1 reports mean and standard deviations by disease groups. All analyzed blood markers showed significant mean differences among disease groups. Patients with IDA had the lowest values of RBC and Hb and the highest values of MCV. 

Table 2 shows the standardized canonical coefficients obtained from the linear discriminant analysis.

MDA analysis for classifying patients into four groups (classification type I) illustrated that two canonical discriminant functions 1 and 2 cumulatively accounted for 99.85% of the total variance (*P* < 0.001 for both functions). In the first function, RBC was negatively correlated with the first function. The rest of variables (Hb, MCV, and MCH) showed negative and significant standardized loadings for the second function.

When classifying patients into two groups (genetic anemia and acquired iron deficiency anemia, classification type II), only one function was needed. In this case, RBC was positively correlated to the discriminant function. 

**Figure 1:**
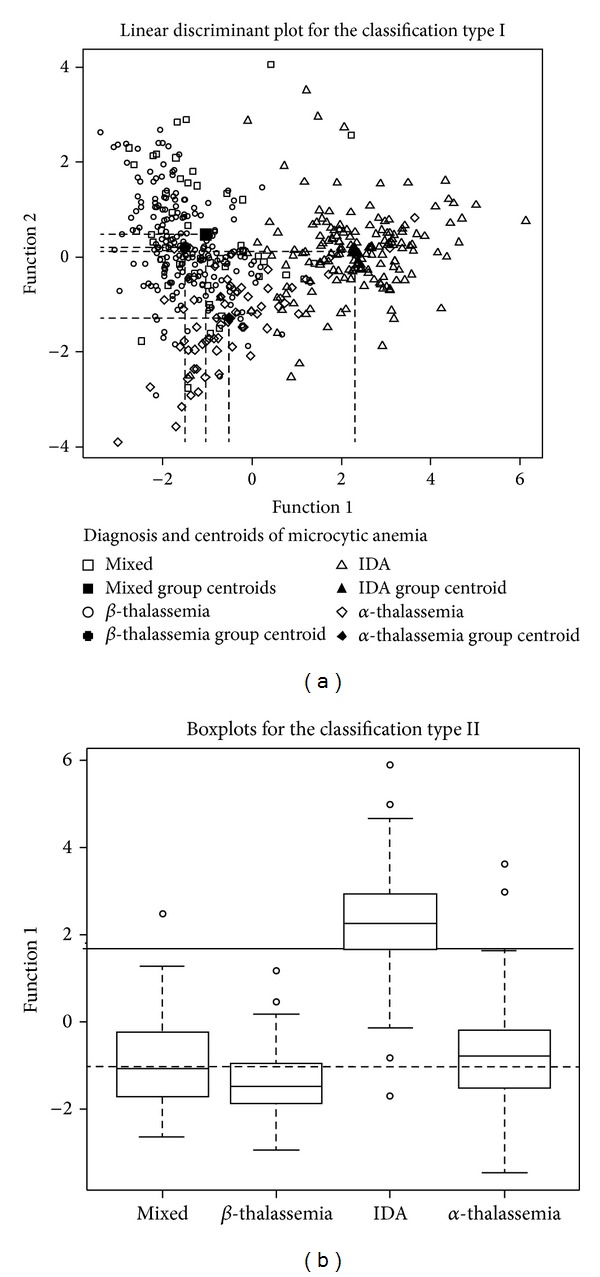
Linear discrimination plot for the studied classification type I (a) and boxplot for the classification type II in the training set (b). Black symbols in the linear discriminant plot indicate centroid groups. Dashed line in the boxplot reflects the cut-off value for the required discriminant function.

Figure 1 shows the linear discriminant plot for the classification type I and the boxplot according the function obtained for the classification type II. In the discriminant plot, there is a significant overlap in the classes corresponding to diseases targeted as *α*- and *β*-thalassemia and the mixed group of thalassemia carriers, whereas patients with IDA are mostly separated from the others (*P* < 0.001). When classifying diseases as acquired or genetic anemia, again there is a clear separation between both blood disorders. 

Once linear discriminant functions were calculated according to the results showed in Table 2, we computed the correct classification rates in the validation set for the entire samples and stratified by disease group.

Tables 3 and 4 display the obtained results. As one can observe in the validation set, when classification type I is applied, 70.3% of IDA disorders were correctly classified, and also *α*-thalassemia had a high rate of correct classification (68.8%); on the other hand only one-third of *β*-thalassemia was recognised, with 39.7% classified as *α*-thalassemia; also one-third of the mixed group of genetic anemia was included in the IDA group.

When trying to classify diseases into two groups (genetic anemia versus IDA, classification type II), the overall rate surpassed the 85% rate (87.9% *β*-thalassemia carriers and 83.3% *α*-thalassemia carriers).

Nineteen % of the patients with genetic anemia were misclassified. Out of these patients, 5 (20.8%) were *β*-thalassemia carriers and 4 (16.7%) *α*-thalassemia, and 15 (62.5%) were mixed group (5 *β*-thalassemia and IDA, 5 *α*-thalassemia and IDA, 1 pregnant *α*-thalassemia, 2 Hb S, and 2 pregnant HbS). 

## 4. Discussion

The screening of thalassemia carriers in endemic areas remains a daily challenge for laboratory professionals. Although thalassemia is most frequent in the Mediterranean basin and Far East countries, due to migration of populations, there is virtually no country in the world now in which thalassemia does not affect some percentage of the inhabitants [7].

On the basis of classical hematological parameters, subjects with IDA are inappropriately discriminated from subjects with anemia due to thalassemia or chronic disease. Some indices have been defined to quickly discriminate both diseases based on the red cell parameters obtained from automated blood cell analyzers and are used as a preliminary screening, with matter of great interest in geographic areas where nutritional deficiencies and thalassemia are present with high prevalence [8]. 

There has been a clear revival of interest in the detection of thalassemia demonstrated by the increasing number of publications reporting new indices in recent years [9–12].

These cell counter-based formulae have been used in the differential diagnosis of microcytic anemia and *β*-thalassemia detection, but when applied to the detection of *α*-thalassemia, or in case of thalassemia and concomitant iron deficiency, these formulae perform much less accurately. 

Another approach to assist in classification of anemia has been the use of computer based expert system subset of artificial intelligence; mimicking the human expert the system applies decision trees, logic rules, or statistical best fit analysis to reach conclusions [13–16].

MDA approach fits fine with the realistic situation a mixed population. An advantage is the simplicity of application; once calculated, the formulae can be incorporated into a programmable calculator or computer spreadsheet, allowing insertion of the hemogram data of certain patients to obtain the provisional classification.

Eldibany et al. [17] applied MDA and identified MCH, RBC count, MCV, and RDW, the best set of indices for differentiating 4 diagnoses. The study demonstrated that a set of linear discriminant functions based on routine hemogram data can effectively differentiate between *α*-thalassemia, *β*-thalassemia, and IDA, with a high degree of accuracy.

As Eldibany et al. proposed, we started the classification type I (Table 2) with two functions and four outcomes, but the results obtained were poorer than those expected, mainly in case of *β*-thalassemia (30% correctly identified). The correct classification for *α*-thalassemia in both studies was around 70%, a high rate that could be taken into account in endemic areas. In the mixed group 29.5% were misclassified as IDA, so 71.5% was recognized as genetic anemia. 

Nevertheless, the HbA_2_ analysis is the gold standard in the diagnosis of thalassemia. The increase of HbA_2_ is the most relevant diagnostic characteristic of *β*-thalassemia carriers and is low or within reference range in *α*-thalassemia patients [18].

We tried to improve the diagnostic performances and the predictor parameters selected, RBC, Hb, MCV, MCH, and RDW, which were used in one function with only two outcomes: acquired anemia (IDA) and genetic anemia. 

The results improved, and 80.5% with genetic anemia were detected, 87.9% *β*-thalassemia carriers, 83.3% *α*-thalassemia carriers, and 72.1% in the mixed group were correctly classified, so we propose a diagnostic based on MDA and HbA_2_ analyses.

The samples classified in the latter group by MDA are selected for HbA_2_ measurement; based on the values obtained and the presence or not of Hb variants, molecular analysis can be performed, but the results obtained in the mixed group suggest that the mixed thalassemia and iron deficiency status remain the most difficult to detect, and 19.5% of the patients with genetic anemia were misclassified as IDA.

It is difficult to talk about thalassemia globally since the social situation and the health systems are diverse anywhere in the world. In the developing countries, where these diseases are endemic, represent a problem of public health, but, in the developed countries with the general budgetary reductions, the presumptive identification of hemoglobin disorders must rely on inexpensive methods of detection, to allow an efficient use of the resources: a good system for screening can help, allowing efficiently selecting samples for further analysis to confirm the disease.

## 5. Conclusions

The above-described system is aimed at screening for thalassemia in samples for which full blood count parameters have been technically and clinically validated prior to the interpretive process. Its main aim is to focus attention and efforts on those samples requiring further investigation for a complete diagnosis.

In an era where demands on laboratories are ever increasing and funding and staffing levels are generally below the desired level, the implementation of a system which reduces staff time and improves result turnaround times is greatly desired. The implementation of a system such as the one we have described will introduce a safe and cost-effective method to minimize the amount of time specialized biomedical scientist spent on analysing results in which no abnormalities are present.

A drawback of this study is the fact that only heterozygous carries were included; the reason was the low prevalence of hemoglobinopathies in our area; perhaps other authors may consider the new approach and would try to verify our findings in areas of high prevalence of these diseases.

## Figures and Tables

**Table 1:** Hematological and biochemical data for the study patients comprised 170 with iron deficiency anemia (IDA), 200 *β*-thalassemia carriers, 65 *α*-thalassemia carriers, and 45 mixed clinical situations (hemoglobinopathy and other disease, iron deficiency, or pregnancy). Values are reported as mean (standard deviation).

	Mixed^a^	*β*-thalassemia^b^	IDA^c^	*α*-thalassemia^d^	*P* value
RBC, 10^12^/L	5.70	5.79	4.72	5.40	<0.001
	(0.57)^c,d^	(0.54)^c,d^	(0.48)^all^	(0.55)^all^	
Hb, g/L	116	119	105	123	<0.001
	(13.2)^d^	(11.2)^d^	(11.0)^c,d^	(14.0)^all^	
MCV, fL	64.6	64.6	73.7	70.5	<0.001
	(4.08)^c,d^	(3.39)^c,d^	(4.63)^all^	(2.96)^all^	
MCH, pg	20.4	20.6	22.3	22.7	<0.001
	(1.48)^c,d^	(1.11)^c,d^	(1.86)^a,b^	(1.08)^a,b^	
RDW, %	16.9	16.1	18.2	15.9	<0.001
	(1.84)^c^	(1.06)^c^	(3.03)^all^	(1.51)^c^	

RBC: Red Blood Cells; Hb: hemoglobin; MCV: mean cell volume; MCH: mean cell hemoglobin; RDW: RBC distribution width.

Superscript letters (^a,b,c,d,all^) indicate significant differences between groups.

*P* < 0.001 was for the studied blood markers for the mean differences between acquired (IDA) and genetic anemia.

**Table 2:** Standardized canonical coefficients obtained from the linear discriminant analysis.

	Classification type I				Classification type II	
	First function		Second function		First function	
	Standardized coefficient	Relative importance	Standardized coefficient	Relative importance	Standardized coefficient	Relative importance
RBC	0.902	−0.532*	5.481	0.154	1.778	0.537*
Hb	−1.362	−0.332	−5.723	−0.410*	−2.225	0.284
MCV	1.607	0.609	0.481	−0.636*	1.814	−0.361*
MCH	−0.257	0.292	2.408	−0.788*	0.152	−0.529*
RDW	0.461	0.267	0.064	0.268	0.441	0.237

Proportion of trace (%)	91.26		8.59		100	

RBC: Red Blood Cells; Hb: hemoglobin; MCV: mean cell volume; MCH: mean cell hemoglobin; RDW: RBC distribution width.

First function: first linear discrimination function. Second function: second discrimination function. Classification type I: disease groups categorized into four diseases: mixed, *β*-thalassemia, *α*-thalassemia, and IDA. Classification type II: targeted diseases as genetic anemia (mixed, *β*- and *α*-thalassemia) and acquired anemia.

Standardized coefficient: standardized coefficient obtained from the linear discriminant analysis of each blood marker for the considered functions.

Relative importance: correlations of each variable with each discriminant functions.

*A correlation higher than 0.40 is considered significant.

Proportion of trace (%): proportion of variability of the outcome explained by the considered independent variables.

**Table 3:** Distribution of predicted versus actual disease classification in the validation set applying the classification type I. Number of patients (column %).

Predicted diagnosis	Actual diagnosis		IDA	*α*-thalassemia	Total
	Mixed	*β*-thalassemia			
Mixed	6 (13.7)	17 (27)	13 (2.6)	2 (12.5)	38
*β*-thalassemia	3 (6.8)	19 (30.1)	3 (0.6)	0	25
IDA	13 (29.5)	2 (3.2)	355 (70.3)	3 (18.7)	373
*α*-thalassemia	22 (50)	25 (39.7)	134 (26.5)	11 (68.8)	192

Total	44	63	505	16	

IDA: iron deficiency anemia.

**Table 4:** Distribution of predicted versus actual disease classification in the validation set applying the classification type II. Number of patients (column %).

Predicted diagnosis	Actual diagnosis	
	Acquired anemia (*n* = 505)	Genetic anemia (*n* = 123)
Acquired anemia, IDA (*n* = 436)	412 (81.6)	24 (19.5)
Genetic anemia (*n* = 192)	93 (18.4)	99 (80.5)

IDA: iron deficiency anemia.
